# Exploring the Role of Presepsin in the Prediction of Atrial Fibrillation Recurrence: Results from the PLACEBO Study

**DOI:** 10.3390/diseases13100349

**Published:** 2025-10-20

**Authors:** Aristi Boulmpou, Christodoulos Papadopoulos, Theocharis Koufakis, Kalliopi Kotsa, Dimitrios Kouroupis, Georgios Dimakopoulos, Aikaterini Balaska, Georgios Zormpas, Michael Doumas, Vassilios Vassilikos

**Affiliations:** 1 Third Department of Cardiology, Aristotle University of Thessaloniki, Ippokratio General Hospital, 54 642 Thessaloniki, Greece; 2 Second Propaedeutic Department of Internal Medicine, Aristotle University of Thessaloniki, Ippokratio General Hospital, 54 642 Thessaloniki, Greece; 3 First Department of Internal Medicine, Aristotle University of Thessaloniki, AHEPA University Hospital, 54 636 Thessaloniki, Greece; 4 Biostats, Epirus Science and Technology Park Campus, University of Ioannina, 45 500 Ioannina, Greece; 5 Second Department of Cardiology, Aristotle University of Thessaloniki, Ippokratio General Hospital, 54 642 Thessaloniki, Greece

**Keywords:** atrial fibrillation, arrhythmia, presepsin, biomarkers, echocardiography, prognosis

## Abstract

Background: Presepsin, a soluble CD14 subtype released during immune activation, has emerged as a marker of inflammation in cardiometabolic disorders. Given the links between inflammation, metabolic dysregulation, and atrial fibrillation (AF), presepsin may represent a novel biomarker for predicting AF recurrence. Aims: To evaluate whether presepsin levels, alone or in combination with other biomarkers and clinical parameters, are associated with paroxysmal AF (PAF) recurrence in a well-characterized cohort from the PLACEBO trial. Methods: This was a retrospective exploratory substudy of 62 patients from the PLACEBO cohort with available baseline presepsin measurements. All patients had a history of PAF and were in sinus rhythm at inclusion. Presepsin and other biomarkers were measured at baseline. Reduced multivariable Cox regression models, limited to two or three predictors, were constructed to avoid overfitting. Results: During 12 months of follow-up, 27 patients (43.5%) experienced AF recurrence. Across the reduced multivariable models, each containing a limited number of predictors, obstructive sleep apnea (OSA) consistently emerged as an independent predictor (HR 3.13–3.36, *p* < 0.05). The use of HR ranges reflects the inclusion of these variables in more than one model. Galectin-3 (GAL3) and standard deviation of R-R intervals (SDRR) did not retain statistical significance, and presepsin was not independently associated with recurrence (HR 1.00, 95% CI 0.92–1.10, *p* = 0.94). Conclusions: In this exploratory study, OSA emerged as the only independent predictor of AF recurrence. Presepsin was not significantly associated with recurrence in the present cohort; however, given the limited sample size and exploratory design, these results do not exclude a potential association. Larger, adequately powered studies are needed to clarify the role of presepsin in AF recurrence risk.

## 1. Introduction

Atrial fibrillation (AF) is the most common cardiac arrhythmia worldwide associated with severe complications, increased mortality and disability rates, also having a negative impact on patient quality of life [1,2]. AF is characterized by a complex pathophysiological background; electric and structural remodeling, autonomic instability, metabolic stress, inflammation, and paracrine effects of epicardial adipose tissue are believed to play a key role [3]. In recent years, the contribution of gut dysbiosis to the development and persistence of AF has been increasingly recognized [4]. Although the underlying mechanisms are still poorly understood, it is postulated that the imbalance in the intestinal flora increases the production of metabolites that exert pro-inflammatory actions resulting in tissue-damaging effects [5]. Furthermore, intestinal dysbiosis is involved in the pathogenesis of other disorders that represent important risk factors for AF, including diabetes, obesity, hypertension, obstructive sleep apnea (OSA), coronary artery disease (CAD), and heart failure (HF) [6,7].

Presepsin is a recently discovered marker of inflammation and sepsis. It is a soluble fragment derived from the N-terminal of an anchored glycoprotein known as CD14, which is present in the membranes of cells that participate in sepsis pathways, including granulocytes, monocytes, and macrophages, acting as a receptor for bacterial lipopolysaccharide (LPS) [8]. Presepsin contributes to the generation of intracellular signals that activate genes involved in the regulation of immune mediators, including cytokines such as tumor necrosis factor-α, interferon, and interleukins, as well as various cell types that perform effector functions [9]. Interestingly, new-onset AF has been frequently observed in critically ill patients with sepsis [10,11], highlighting the existence of common pathogenetic pathways between the two entities in which systemic inflammation plays a predominant role. In people with cardiometabolic disorders, gut dysbiosis causes a disruption of the intestinal barrier, leading to the penetration of gut-origin LPS into the systematic circulation even in the absence of infection, a phenomenon known as “metabolic endotoxemia” [12]. Given that presepsin is a component of the LPS receptor, it has been proposed as an emerging biomarker in cardiometabolic diseases, reflecting the magnitude of both inflammation and intestinal dysbiosis [13]. In fact, several studies have found increased circulating presepsin levels in infection-free individuals with metabolic disorders, including diabetes and obesity, compared to controls [14,15].

There is no doubt that predicting new-onset or recurrent AF is of great clinical importance due to the potential to prevent serious adverse events, such as embolic stroke, HF, and even death [16]. Until now, numerous biomarkers have been investigated as possible predictors of AF, including high-sensitivity cardiac troponin I (hs-cTnI), natriuretic peptides, and C-reactive protein (CRP) [17]. Given the important limitations of biomarkers as predictive tools, such as different cut-off points and variable sensitivity and specificity according to the population applied, other studies attempted to combine biochemical and echocardiographic parameters to enhance predictive ability [18,19]. Although presepsin has been previously evaluated as a predictor of major adverse cardiovascular (CV) events in patients at high CV risk who underwent non-cardiac surgery [20], there is a knowledge gap regarding its predictive value in AF.

To our knowledge, no prior study has directly evaluated presepsin in relation to AF onset or recurrence. As mentioned above, existing AF literature implicates inflammatory pathways and monocyte activation (including CD14-related markers), whereas presepsin has mainly been studied in sepsis and cardiometabolic contexts without AF endpoints. Therefore, the aim of this pilot study was to investigate whether the evaluation of presepsin levels, alone or in combination with other plasma biomarkers, echocardiographic indices, and cardiopulmonary exercise testing (CPET) parameters, contributes to the prediction of paroxysmal AF (PAF) recurrence. This report builds upon the previously published PLACEBO trial, which integrated echocardiographic indices, autonomic markers, and biomarkers, to predict PAF recurrence, by specifically analyzing a subgroup of patients in whom presepsin levels were additionally measured [21].

## 2. Materials and Methods

### 2.1. Study Design and Population

This was a retrospective exploratory analysis conducted within the context of the prospective PLACEBO trial (ClinicalTrials.gov Identifier: NCT05246423), a single-center, observational cohort study carried out at Ippokratio General Hospital in Thessaloniki, Greece. The study adhered to the Declaration of Helsinki and received approval from the local ethics committee (approval no. 375/30-6-20). All patients provided written informed consent, including permission for the use of stored biological samples. The detailed study protocol has been reported elsewhere [22].

For this analysis, we evaluated a subset of 62 random patients from the PLACEBO cohort for whom preserved blood samples were available for presepsin measurement. All patients were in sinus rhythm at baseline and met the original study’s inclusion and exclusion criteria. Patients were followed prospectively for 12 months for the detection of PAF recurrence, defined as any episode lasting ≥ 30 s, identified through 24 h Holter monitoring, documentation in medical records, or verified patient-reported symptomatic episodes managed as AF.

### 2.2. Data Collection and Parameters

Baseline assessment included demographic and clinical data, echocardiographic parameters such as right ventricular fractional area change (RV FAC), and heart rate variability metrics obtained from 24 h Holter monitoring, including the standard deviation of RR intervals (SDRR). OSA was documented based on a previously established diagnosis. Measured plasma biomarkers included galectin-3 (GAL3), high-sensitivity cardiac troponin I (hs-cTnI), brain natriuretic peptide (BNP), and presepsin.

Presepsin was determined by a sandwich enzyme-linked immune sorbent assay (ELISA) method (FineTest Human Presepsin ELISA kit, Wuhan Fine Biotech Co., Ltd., Wuhan, China). Anti-presepsin antibody and biotin-conjugated detection anti-presepsin antibody were used. Horseradish peroxidase (HRP)-streptavidin and 3,3′,5,5′-Tetramethylbenzidine (TMB) substrate were added to visualize the HRP enzymatic reaction. The absorbance was read at 450 nm in a microplate reader (Biobase Co., Ltd., Jinan, China). The concentration of presepsin in the serum was calculated by drawing a standard curve. The concentration was proportional to the OD450 value. The Inter-Assay CV was <10% and the Intra-Assay CV was <8%.

### 2.3. Outcome and Follow-Up

The primary endpoint was time to first documented recurrence of PAF within 12 months of baseline evaluation. AF recurrence was defined as any confirmed episode lasting at least 30 s, identified via scheduled 24 h Holter ECG recordings, review of medical records, or verified patient-reported symptomatic episodes.

### 2.4. Statistical Analysis

Continuous variables were tested for normality using the Shapiro–Wilk test and are presented as mean ± standard deviation or median (interquartile range) as appropriate. Categorical variables are expressed as absolute counts and percentages. Between-group comparisons for continuous variables were performed using the independent samples *t*-test or the Mann–Whitney U test, as appropriate. Categorical variables were compared using the χ^2^ test or Fisher’s exact test.

Time-to-event analyses were conducted using Cox proportional hazards regression. The primary outcome was time from baseline to first documented AF recurrence within one year. In line with established statistical guidelines for small sample sizes, the number of predictors in multivariable models was limited to ensure at least 10 events per variable to reduce the risk of overfitting.

Before constructing multivariable models, we performed a complete univariable Cox regression analysis for all prespecified clinical and biomarker variables [OSA, hypertension, diabetes, chronic obstructive pulmonary disease (COPD), stroke, thyroid disease, b-blocker/antiarrhythmic therapy, GAL-3, SDRR, and presepsin]. To improve interpretability and address scale concerns, presepsin was additionally modeled (i) per 0.1 ng/mL, (ii) per 1 standard deviation, and (iii) on the log-transformed scale. Candidate variables for multivariable modeling were selected on the basis of both clinical judgment and univariable evidence (*p* < 0.10). Multicollinearity was assessed using Spearman correlation coefficients and variance inflation factors (VIF > 5 as threshold); all VIFs were ~1.0 and |ρ| ≤ 0.14, indicating no collinearity. To minimize overfitting given the limited number of events, reduced (parsimonious) multivariable Cox models including 3 or less predictors were constructed, with presepsin forced into each model due to the study focus. These reduced models were selected a priori based on the strongest univariable associations and clinical relevance, including presepsin, OSA, GAL3, and SDRR, and in line with the already established prognostic significance of these parameters in the primary PLACEBO trial [21].

Results are reported as hazard ratios (HR) with 95% confidence intervals (CI). A two-tailed *p*-value < 0.05 was considered statistically significant. Analyses were performed using R version 4.4.3.

## 3. Results

A total of 62 patients with available presepsin measurements were included in the analysis. The study population was relatively homogeneous in terms of demographic and clinical characteristics, including age, comorbidities, and antiarrhythmic drug use (Table 1). Baseline demographic and clinical characteristics of the selected subgroup did not differ significantly from those of the overall PLACEBO cohort, indicating that the present sample is representative of the parent population. All patients had a history of PAF and were in sinus rhythm at baseline, with preserved left ventricular function and no evidence of acute illness or decompensated heart failure. Most participants were clinically stable, and there was a low burden of structural heart disease, allowing for a focused evaluation of inflammatory and autonomic predictors.

Regarding comorbid conditions, hypertension and dyslipidemia were the most prevalent (45.2% and 49.3%, respectively), with lower rates of diabetes mellitus, COPD, and CAD. However, no statistically significant differences in these variables were observed between recurrence and non-recurrence groups, suggesting a relatively homogeneous sample in terms of baseline comorbidity burden.

During the 12-month follow-up, 27 patients (43.5%) experienced AF recurrence. We first performed univariable Cox regression analyses for all prespecified clinical and biomarker variables to identify potential predictors of AF recurrence. As shown in Table 2, OSA was significantly associated with recurrence (HR 3.12, 95% CI 1.02–9.56; *p* = 0.047), whereas arterial hypertension, diabetes mellitus, GAL-3, SDRR, and presepsin were not (all *p* > 0.30). These results informed the construction of reduced multivariable models, as detailed below.

Variables showing potential associations in univariable analyses (*p* < 0.10) and clinically relevant factors were subsequently included in reduced multivariable Cox models (Table 3). In the reduced multivariable models, OSA remained the only statistically significant predictor of AF recurrence. For example, in the model including OSA and GAL-3, only OSA was significant (HR 3.31, 95% CI 1.06–10.31, *p* = 0.039). When presepsin was included alongside OSA, only OSA was significant (HR 3.13, 95% CI 1.01–9.68, *p* = 0.047), while presepsin was not (HR 1.00, 95% CI 0.92–1.10, *p* = 0.941).

In an additional exploratory multivariable model including OSA, hypertension, and presepsin, selected on the basis of univariable and clinical relevance, hypertension remained significantly associated with AF recurrence (HR 3.32, 95% CI 1.14–9.65; *p* = 0.028), whereas presepsin was not (HR 1.01, 95% CI 0.93–1.11; *p* = 0.79). Respecifying presepsin per 0.1 ng/mL, per 1 SD, or on the log scale did not materially alter these findings (all *p* > 0.80).

## 4. Discussion

In this retrospective exploratory substudy conducted within the prospective PLACEBO trial, we evaluated presepsin as a potential biomarker of AF recurrence using reduced Cox regression models tailored to the modest sample size. Across all specifications, OSA consistently emerged as an independent predictor, whereas GAL-3 and SDRR did not retain statistical significance in this subset. Presepsin, despite its mechanistic plausibility as a marker of subclinical inflammation and metabolic stress, was not independently associated with AF recurrence. These findings indicate that, in this limited cohort, established predictors such as OSA outweighed presepsin in prognostic importance. Larger, adequately powered studies are needed to clarify whether presepsin provides incremental prognostic value beyond conventional markers.

Presepsin is a soluble CD14 subtype released in response to bacterial LPS and reflects innate immune activation [23]. While originally studied in the context of sepsis, recent evidence has expanded its relevance to chronic inflammatory conditions and cardiometabolic disease, where low-grade endotoxemia and immune dysregulation contribute to vascular and myocardial remodeling [9,24]. Although presepsin levels were not independently associated with AF recurrence in this study, their established role as markers of innate immune activation supports further investigation into the potential contribution of low-grade inflammation to arrhythmia persistence. Rescaling presepsin values and re-evaluating its effect in full univariable and clinically guided multivariable models did not change the inference, further supporting the robustness of this finding.

The established role of presepsin as a marker of innate immune activation suggests a potential link between systemic inflammation and arrhythmia persistence, despite the non-significant association of this biomarker with AF recurrence in this study. This observation, in line with previous evidence implicating inflammatory and metabolic pathways in AF pathophysiology, supports further evaluation of presepsin in larger and longitudinal cohorts. However, the absence of an observed association in our cohort should be interpreted with caution, as the limited sample size substantially reduces statistical power and may obscure modest effects. Therefore, these findings are best viewed as inconclusive rather than definitively negative.

Systemic and subclinical inflammation are known contributors to AF vulnerability, and presepsin may be involved in this pathway [25]. In the same line, presepsin, as a component of the CD14 receptor complex involved in LPS recognition, may reflect low-grade immune activation driven by gut-derived endotoxemia or chronic metabolic dysregulation [13]. This mechanistic pathway is increasingly recognized as a contributor to atrial remodeling and arrhythmogenesis [26,27]. Unlike conventional biomarkers such as CRP, which reflect generalized systemic inflammation, or natriuretic peptides, which primarily relate to myocardial stretch, presepsin may capture a distinct inflammatory pathway involving immune signaling, microbial translocation, and tissue-level immune activation [28,29]. Its elevation in metabolically affected but infection-free individuals suggests that presepsin may serve as a sensitive and specific biomarker for a subgroup of AF patients with underlying low-grade inflammation [30]. Future studies should clarify its incremental value in risk stratification and define clinically relevant cut-offs.

OSA emerged as the strongest and most consistent determinant of AF recurrence in our cohort. The persistent association across all models underscores the well-established contribution of OSA to AF pathophysiology through intermittent hypoxia, autonomic imbalance, and systemic inflammation [31,32]. These findings highlight the clinical relevance of systematic screening and management of OSA as an integral part of AF care, as effective treatment may reduce arrhythmia burden and improve rhythm control outcomes. GAL-3 and SDRR, although prognostic in the primary PLACEBO trial [21], were not significant in this substudy, likely reflecting the limited statistical power rather than a true absence of association.

We also conducted a comprehensive univariable screening and an additional clinically guided multivariable analysis including arterial hypertension, OSA, and presepsin. Although arterial hypertension emerged as a significant predictor, presepsin remained non-significant even after rescaling and log transformation, supporting the robustness of our findings. The absence of multicollinearity among candidate predictors further strengthens model stability.

The above findings should be interpreted in the context of the study’s limitations. This was a retrospective, exploratory analysis with a modest sample size, and presepsin was measured at a single time point. Furthermore, the lack of continuous monitoring may have underestimated the true recurrence rate, particularly for asymptomatic episodes, whilst AF recurrence may have been misclassified in some cases due to reliance on patient-reported symptoms. However, this strategy reflects real-world clinical practice and was supported by documentation or physician confirmation when possible. Additionally, while the relatively homogeneous clinical profile of our cohort may have reduced the impact of major confounding factors, the possibility of residual confounding from unmeasured variables cannot be excluded. Finally, while antiarrhythmic and beta-blocker use was recorded and analyzed, other medications that may influence autonomic tone were not systematically recorded, representing another limitation of the study.

Despite these limitations, the study offers important strengths. It introduces presepsin into AF research for the first time and examines it alongside validated predictors such as OSA and GAL3. The integration of inflammatory, autonomic, and structural parameters reflects the multifactorial nature of AF recurrence [33,34,35]. Although presepsin was not independently predictive in this analysis, it remains a biologically plausible marker and may add value in combination with other parameters in larger, prospective studies.

## 5. Conclusions

In this retrospective exploratory substudy, OSA emerged as a strong and independent predictor of AF recurrence. In contrast, presepsin did not demonstrate independent prognostic value for arrhythmia recurrence in our reduced multivariable models. While presepsin was not significantly associated with outcomes in this limited sample, its potential role as a biomarker remains of interest. Future larger, prospective studies are warranted to further evaluate the utility of presepsin in risk stratification and management of patients with AF.

## Figures and Tables

**Table 1:** Baseline demographic and clinical characteristics of the study population according to AF recurrence during follow-up. Categorical variables are presented as number (%) and compared using the Chi-square (χ^2^) test. Categorical variables are presented as number (%) and were compared using the Chi-square (χ^2^) test or Fisher’s exact test, as appropriate. Continuous variables are presented as median (interquartile range) and were compared using the Mann–Whitney U test. No statistically significant differences were observed between patients with and without AF recurrence in terms of gender, smoking status, alcohol consumption, antiarrhythmic therapy, beta-blocker use, BMI, or disease duration. Abbreviations: AF, atrial fibrillation; COPD, chronic obstructive pulmonary disease; BMI, body mass index; SD, standard deviation; MW, mean weighted.

	New AF Episode							
	No		Yes		Chi Square			
	N	%	N	%	X_I_^2^	*p*-Value		
Female gender	24	57.1%	14	45.2%	1.026	0.311		
Smoking	14	33.3%	9	29.0%	0.153	0.696		
Exercise	27	64.3%	23	74.2%	0.811	0.368		
Alcohol	20	47.6%	17	54.8%	0.372	0.542		
Dyslipidemia	20	47.6%	16	51.6%	0.114	0.736		
Arterial hypertension	18	42.9%	15	48.4%	0.220	0.639		
Diabetes mellitus	3	7.1%	3	9.7%	0.152	1.000		
COPD	2	4.8%	1	3.2%	0.107	1.000		
Heart failure	0	0.0%	1	3.2%	1.374	0.425		
Coronary artery disease	2	4.8%	1	3.2%	0.107	0.744		
Valvular disease	3	4.8%	0	0.0%	2.309	0.257		
Stroke	0	0.0%	2	6.5%	2.786	0.177		
Thyroid disease	11	26.2%	6	19.4%	0.467	0.582		
Antiarrhythmic drugs	18	42.9%	16	51.6%	0.550	0.459		
B-blocker	28	66.7%	18	58.1%	0.566	0.452		
B-blocker & antiarrhythmic drugs	16	38.1%	13	41.9%	0.110	0.740		
	M (SD)	N	M (SD)	N	M (SD)	N	MW	*p*-value
Age (years)	58.7 (13.1)	42	60.8 (9.4)	31	59.6 (11.6)	73	654.5	0.969
BMI	28.2 (4.6)	42	27.4 (4.1)	31	27.9 (4.4)	73	591.5	0.507
Total disease duration (months)	38.6 (54.3)	42	41.9 (42.6)	31	40.0 (49.4)	73	739	0.325

**Table 2:** Univariable Cox regression results for AF recurrence. Hazard ratios (HR) and 95% confidence intervals (CI) were estimated using univariable Cox proportional hazards models. Binary variables were coded as 0 = absent, 1 = present. Continuous variables were analyzed as linear predictors. Abbreviations: OSA, obstructive sleep apnea; COPD, chronic obstructive pulmonary disease; GAL-3, galectin-3; SDRR, standard deviation of R-R intervals. N/A: model did not converge due to low event frequency.

Variable	Hazard Ratio (HR)	95% CI Lower	95% CI Upper	*p*-Value
OSA	3.12	1.02	9.56	0.047
Arterial hypertension	1.40	0.64	3.05	0.401
Diabetes mellitus	1.10	0.32	3.76	0.874
COPD	N/A	N/A	N/A	Singular matrix
Stroke	1.24	0.16	9.37	0.836
Thyroid disease	2.49	0.80	7.73	0.115
GAL-3 (ng/mL)	1.05	0.93	1.17	0.443
Presepsin (ng/mL)	1.00	0.91	1.09	0.923
SDRR	1.00	0.97	1.02	0.852

**Table 3:** Multivariable Cox regression analysis for AF recurrence (reduced models). Hazard ratios (HRs) and 95% confidence intervals (Cis) are from reduced Cox regression models (n = 28). Statistically significant predictors are shown in bold. Abbreviations: OSA, obstructive sleep apnea; GAL3, galectin-3; SDRR, standard deviation of RR intervals.

Model Predictors	Variable	Hazard Ratio (HR)	95% CI	*p*-Value
Model 1 (OSA + GAL3)	OSA	3.31	1.06–10.31	0.039
	GAL3 (ng/mL)	1.06	0.94–1.19	0.348
Model 2 (OSA + GAL3 + SDRR)	OSA	3.36	1.08–10.49	0.037
	GAL3 (ng/mL)	1.06	0.94–1.20	0.323
	SDRR	0.99	0.97–1.02	0.690
Model 3 (OSA + presepsin)	OSA	3.13	1.01–9.68	0.047
	Presepsin (ng/mL)	1.00	0.92–1.10	0.941

## Data Availability

The data presented in the study are available on request from the corresponding author. The data are not publicly available due to privacy restrictions of the Greek National Health System.

## Abbreviations

The following abbreviations are used in this manuscript:

AF	atrial fibrillation
OSA	obstructive sleep apnea
CAD	coronary artery disease
COPD	chronic obstructive pulmonary disease
HF	heart failure
LPS	lipopolysaccharide
Hs-cTnI	high-sensitivity cardiac troponin I
CRP	C-reactive protein
CV	cardiovascular
CPET	cardiopulmonary exercise testing
PAF	paroxysmal atrial fibrillation
RV FAC	right ventricular fractional area change
SDRR	standard deviation of RR intervals
GAL3	galectin-3
ELISA	enzyme-linked immune sorbent assay
HRP	horseradish peroxidase
TMB	tetramethylbenzidine
HR	hazard ratio
CI	confidence interval
SD	standard deviation
BMI	body mass index
